# Hippocampal Neurochemical Changes in Senescent Mice Induced with Chronic Injection of D-Galactose and NaNO_2_: An *In Vitro* High-Resolution NMR Spectroscopy Study at 9.4T

**DOI:** 10.1371/journal.pone.0088562

**Published:** 2014-02-12

**Authors:** Yan Lin, Jianli Yao, Yaowen Chen, Li Pang, Haihong Li, Zhen Cao, Kezeng You, Haiyang Dai, Renhua Wu

**Affiliations:** 1 Department of Medical Imaging, The Second Affiliated Hospital, Shantou University Medical College, Shantou, Guangdong Province, China; 2 Shantou University Central Laboratory and NMR Unit, Shantou, Guangdong Province, China; 3 Mental Health Center of Shantou University Medical College, Shantou, Guangdong Province, China; 4 Key Laboratory of Molecular Imaging of Guangdong Province, Shantou, China; 5 Sichuan Provincial Tumor Hospital, Chengdu, China; University of South Florida, United States of America

## Abstract

Proton magnetic resonance spectroscopy (^1^H-MRS) has been used to provide useful information about the neurochemical changes reflecting early pathological alterations in Alzheimer's disease (AD) brain. In this study, we have longitudinally measured the hippocampal neurochemical profile *in vitro* in senescent mice induced with chronic injection of D-Galactose and NaNO_2_, at different time point from day 30 to day 70 with a 10-day interval. Pathological brain alterations induced by D-Galactose and NaNO_2_ were monitored through hematoxylin and eosin (HE) staining, Congo red staining and bielschowsky silver staining, and the cognition deficits were assessed via Morris Water Maze (MWM) test. This D-galactose and NaNO_2_ treated mouse model, characterized by an early-onset memory dysfunction, a robust neuronal loss, amyloid plaques and neurofibrillary tangles in hippocampal subdivision, well mimics a prodromal Alzheimer's phenotype. Consistent with previously published *in vivo* ^1^H MRS findings in human AD patients and AD transgenic mice, our *in vitro* ^1^H MRS on the perchloric acid extractions of hippocampus in senescent mice observed significant decreases of N-acetylaspartate (NAA) and Glutamate (Glu) but an increase in Myo-inositol (mIns). Elevated mIns occurred prior to the reduction of NAA and Glu during the progression of aging. In addition, changes in mIns, NAA and Glu were found to precede pathological abnormalities. Overall, our *in vitro* findings in senescent mice validated the concept that hippocampal neurochemical alternations preceded the pathological changes of the brain, and could serve as potential markers of AD progression. Reductions of NAA and Glu can be interpreted in terms of neuronal degeneration and dysfunctions in glutamatergic activity that may contribute to the pathophysiological mechanisms underlying AD. Elevated mIns might be related to glial activation. Further experiments are needed to explore the potential value of mIns in the early diagnosis of AD, to verify whether glial cell proliferation occurs earlier than neuronal changes.

## Introduction

Alzheimer's disease (AD), a progressive neurodegenerative disorder, has become a major public health concern in societies with aging populations [1], [2], [3]. Medical treatment is effective only for patients with pre-symptomatic AD and mild cognitive impairment (MCI) in the prodromal stage, who have not yet presented irreversible neuronal changes [4], and only early medical intervention may delay the progression of AD and improve the patient's quality of life. Therefore, there is considerable value to identify disease-specific markers for AD progression which would aid in early diagnosis, drug development and therapeutic monitoring.

Neuroimaging has been used to investigate morphological abnormalities (such as MRI-based volumetric measurement of hippocampus [5], [6], amyloid plaques imaging [7], [8], [9]) and neurochemical alterations [10], [11], [12], [13], [14] in clinical AD brains during disease progressions. AD-related neurochemical alterations were found to precede morphological changes (reviewed in [15]), and were regarded as plausible markers of the pathological progression in AD. Proton magnetic resonance spectroscopy (^1^H-MRS) is a powerful tool for assessing the metabolic and biochemical changes of living tissues as well as quantitative analyses of compounds [16], [17], [18], thus bringing hope for early detection of AD. Among ^1^H MRS-detectable metabolites, N-acetylaspartate (NAA), creatine and phosphate creatine (tCr), choline (Cho), myo-inositol (mIns) and glutamate (Glu) are of particular interest, since they belong to specific neuronal and glial metabolic pathways, membrane constituents, and energy metabolism. Decreased neuronal metabolite NAA and increased glial metabolite mIns have been reported in the ^1^H MR spectra of patients with MCI and early AD in contrast to their levels in the cognitively normal elderly [10], [11], [12], [19], [20], [21], [22], [23]. Disturbances in the levels of neurotransmitter Glu have also been noted [13], [24]. There are controversial reports on the changes in the membrane integrity marker Cho levels. Some studies found elevated Cho levels in the temporal, parietal and occipital lobes of AD patients [14], [22], [25], and others reported no significant changes [26], [27]. Besides these, abnormal levels in Cr, lactate (Lac), taurine (Tau) and g-aminobutyric acid (GABA) were also reported in human AD brain.

Histopathological findings are considered to be the “gold standard” in evaluating surrogate markers for disease progression in AD brain. Measurements of *in vivo* ^1^H MRS on senescent human brain have been performed on clinical AD patients, but studies to verify the correlations of these measurements with the pathological human brain lesions are limited. The use of animal models is a more preferred substitute. Transgenic mouse models, most of which were based on the overexpression of mutated forms of human amyloid-β protein precursor (AβPP) or in combination with mutated human presenilin 1 (PS1) or 2 (PS2) genes [28], reproduced the histopathological features of AD. Studies of different AD transgenic mice *in vivo* have shown decreases in NAA and increases in mIns [29], [30], [31]. However, most of these animal models failed to show the complete phenotype of AD including neurofibrillary tangles and massive neuronal loss [32]. Improvements in mouse models are needed for better representation of human AD [33], [34].

In this study, senescent mice (prodromal stage AD) were induced with chronic injection of D-galactose and NaNO_2_. This senescent mouse model is characterized by an early-onset memory dysfunction and morphological abnormalities including neuronal loss, amyloid plaques and neurofibrillary tangles in hippocampal subdivision, mimicking a prodromal Alzheimer's phenotype. Longitudinal measurements of hippocampal neurochemical alternations in senescent mice at different time point from day 30 to day 70 with a 10-day interval were conducted using *in vitro* high resolution ^1^H MRS at 9.4T. In order to accomplish this, perchloric acid extractions of the hippocampus were collected and *in vitro* ^1^H MRS was performed on the resulting extracts. A greater number of metabolites could be investigated *in vitro* based on high-resolution NMR studies of brain extracts [35], [36], which allows for more homogeneous NMR solution samples and improving metabolites discrimination and quantification. It could be also useful in cases when resolution and SNR of *in vivo* spectra are not sufficient for quantification of some metabolites, in particular those with low concentrations. As expected, hippocampal neurochemical alternations were found to occur prior to brain morphological changes. In good agreement with previous *in vivo* ^1^H MRS findings in human pre-symptomatic AD and AD transgenic mice, our *in vitro* ^1^H MRS in senescent mice observed decreased NAA and Glu and increased mIns. Strong correlation was also observed between the *in vitro* ^1^H MRS findings and the histopathological observations.

## Materials and Methods

### Animals

Thirty six 3-month old Kunming male mice (purchased from the Experimental Animal Center of Shantou University Medical College, China) were housed in cages in an air-conditioned room under controlled temperature (22°C±3°C) and humidity (45%–65%), for 7 days before the experiment. The animals were maintained in a 12 h-light/dark cycle (7 am on-7 pm off), with free access to food and water. They were randomly and equally divided into one control group and five model groups (n = 6). The mice in the model groups were injected with D-galactose (120 mg/kg body weight) and NaNO_2_ (90 mg/kg body weight), once daily for 30 days, 40 days, 50 days, 60 days and 70 days, respectively. While the control mice were administrated with the same volume of saline (210 mg/kg) once daily for 70 days. The injection was started at the same day for all mice. On that day after the final injection, all mice were longitudinally subjected to a series of tasks using the conventional Morris Water Maze (MWM) test to evaluate the hippocampal-dependent spatial learning and memory abilities. After behavioral tests, all mice were sacrificed by cervical dislocation and brains were removed immediately. The left-sided and right-sided hippocampal tissues were immediately collected and frozen in liquid nitrogen and kept at −80°C until use. All experimental protocols were approved by the Ethics Committee of Shantou University Medical College and all experiments were performed in accordance with guidelines from the Chinese Animal Welfare Agency. All efforts were made to minimize animal suffering and to reduce the number of animals used.

### Morris Water Maze (MWM) Test

The MWM test plays an important role in the validation of rodent models for neurocognitive evaluation and results are expressed by escape latencies to find the hidden platform. A standard hidden platform protocol was employed. A circular white pool with a diameter of 120 cm and a depth of 50 cm, was filled with opaque water (25±1°C) to a height of 35 cm. A white escape platform (r = 12 cm) was submerged 1.2 cm below the water surface at a constant position in the center of the North-West (NW) quadrant during training. To reduce stress effects, mice were habituated to the maze 24 hours prior to training. Animals were introduced to the pool from start positions East (E), South-East (SE), South (S), and South-West (SW) to avoid close initial proximity to platform. Each mouse was placed into the pool from quasi-random start points and allowed a maximum of 120 s to escape to the platform. After mounting the platform, animals were left there for 15 s. Animals failing to locate the platform were guided with a wooden rod and also left there for 15 s. This 4-trial training task was repeated for 4 consecutive days with inter-trial intervals (ITI) of 60 minutes. During place navigation training, the ability of mice to learn the location of a fixed hidden platform was assessed by escape latencies. Maze performance was recorded using a video camera suspended above the maze and interfaced with a video tracking system (HVS Imaging, Hampton, UK). The swimming path of each mouse was tracked and analyzed by a computer-based video tracking system (HVS Imaging, Hampton, UK). When testing was complete, the mice were dried off and returned to their housing facility once normothermia was assured.

### *In Vitro* ^1^H MRS Examination

#### 1.1 Preparation for NMR Spectroscopy

The left-sided frozen hippocampus tissues were disrupted using an automated ball mill until a creamy consistency was reached. Wet tissue homogenate was extracted with ice-cold perchloric acid and neutralized with ice-cold KOH (pH = 7). The solvent-tissue mixtures were then vortexed in the glass tubes for 10 minutes and centrifuged for 20 minutes (10,000 rpm), to facilitate the separation of the two superimposed liquid layers. The supernatant was collected and lyophilized, then redissolved in deuterated water (D_2_O). A known concentration of deuterated trimethylsilylproprionate (TSP) was then added to each aqueous sample as an internal standard. A total volume of 0.6 ml solution was transferred into 5 mm diameter NMR tubes.

#### 1.2 *In Vitro* NMR Spectroscopy Acquisition

^1^H NMR spectrum of all hippocampus extracts were obtained with a 9.4T Bruker Avance vertical bore magnet equipped with a Quattro Nucleus Probe (QNP) probe (5 mm dual ^13^C/^1^H probe head) at 25°C. The supporting software consisted of the ICON-NMR 4.2 package for the TopSpin software (v. 2.1; Bruker BioSpin, Germany). Before spectral acquisition, the probe was manually tuned and matched to the ^1^H frequency and locked to ^2^H. The samples were shimmed using the automated ‘topshim’ program provided by the TopSpin software and tuned iteratively in all XYZ-directions afterwards. Spectra of the hippocampus extracts were recorded with water presaturation using a pulse-acquire sequence with the following parameters: central frequency = 400 MHz, number of data points = 32768, spectral width = 8223 Hz, number of averages = 128, relaxation delay = 2 s, dwell time = 60.8 µs, acquisition time = 2.56 s.

#### 1.3. Metabolites Quantification

Postprocessing was carried out semi-automatically using Bruker Topspin 2.1 software. The following procedures were included: (a) Exponential line broadening (3 Hz), (b) zero/first-order phase correction and (c) baseline correction. The chemical shift was assigned according to the internal standard TSP (0.0 ppm) and then advanced analysis was performed using Topspin 2.1. The peak areas which were assigned as metabolites containing NAA, Glu, total Cr, Cho, mIns and lactate (Lac) were integrated using Topspin 2.1 software with baseline flattening around each integration region. The metabolite concentrations were determined as μmol per gram of the amount of wet brain tissue after homogenate, relative to TSP as an internal reference. The following formula was used. [C_i_ = (A_i_/A_TSP_)*(N_TSP_/N_p,i_)*C_TSP_] where C is the concentration, A is the peak area, N_p,i_ is the number of protons contributing to the resonance of metabolite i (i = NAA (N_p_ = 3), Glu (N_p_ = 2), tCr (N_p_ = 5), mIns (N_p_ = 4), Lac (N_p_ = 3), Cho (N_p_ = 9)), N_TSP_ correspond to the number of protons giving rise to the TSP peak.

### Pathomorphology

To confirm the presence of neuropathological alterations induced by D-galactose and NaNO_2_, hematoxylin and eosin (HE) staining [37], Congo red staining [38] and bielschowsky silver staining [39] were performed using standard histological techniques. The right-sided frozon hippocampus tissues were sliced and stained for histopathological examinations. Samples were fixed in 4% paraformaldehyde in 0.01 M PBS (pH 7.4) for 8 hours, dehydrated, and embedded with paraffin. Sections were cut into 5 µm thick pieces, deparaffinized, and then rehydrated in a gradient of high percentage ethanol to distilled water, and were stained with HE for the observation of cell apoptosis, with Congo red for amyloid plaques and with bielschowsky silver for neurofibrillary tangles.

#### 1.1. HE Staining

Deparaffinized and rehydrated sections were washed in distilled water for three times, and stained with the alum haematoxylin for 20 minutes, and then rinsed with running tap water for 5 minutes. They were subsequently immersed in 1 % acid alcohol for 10 seconds, blued up in 1 % Scott's tap water substitute for 10 seconds, and then rinsed in tap water for 5 minutes. The nuclear staining was followed by counterstaining with eosin for 2 minutes, dehydrated through 95 % alcohol, cleared in xylene and mounted with resinous mounting medium for microscopic examination. To evaluate the level of apoptosis, sections were examined under a light microscope (Olympus, Japan). Photos of typical lesion were randomly taken and blindly coded, with 15 fields in consecutive sections of the hippocampus with a 400-fold magnification and a 1×1-mm grid.

#### 1.2. Congo Red Staining

Deparaffinized and rehydrated sections were first stained in Gill's haematoxylin solution (Sigma, stl Louis) for 10 minutes and then rinsed in running tap water for 5 minutes and incubated in alkaline sodium chloride solution for 20 minutes. Sections were then stained in Congo red working solution for 15 minutes (0.2 % in 80 % ethanol saturated with sodium chloride; Sigma), followed by dehydration through 95 % alcohol. They were then dehydrated, hyalinized and mounted for microscopic examination.

#### 1.3. Modified Bielschowsky Silver Staining

Deparaffinized and rehydrated sections were incubated in pre-warmed (37°C) 20 % silver nitrate solution for 25 minutes, washed with distilled water for 3 times, and then immersed in 10 % formalin to stop the silver reaction. The slides were then added with ammonium silver solution drop by drop followed by rinsing in 10 % formalin until the sections became dark brown. They were then washed in distilled water for 5 minutes, placed in 5 % sodium thiosulfate solution for 5 minutes, dehydrated, hyalinized and mounted.

### Statistical Analysis

All results are expressed as mean ± SD. All data were statistically analyzed using SPSS 16.0 statistical software with p<0.05 considered as being statistically significant. The escape latencies in MWM test were analyzed by Repeated Measures and Multivariate Analysis of Variance (ANOVE) process of the general linear model in SPSS. Comparisons of the ^1^H-MRS between the model groups and the control group at each time point were conducted by using a two-sample t-test. Metabolite changes among the model groups during different time points of injection were assessed by means of a one-way ANOVA.

## Results

### Behavioral Test

**Figure 1:**
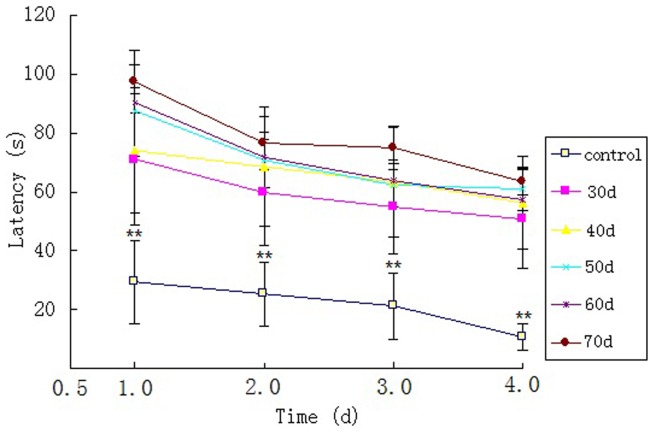
A tendency toward a decreased latency in MWM during place-training trials, while the number of training was increased along the sessions in any group. Data is presented as 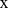
±s (** p<0.01: D-galactose and NaNO_2_ treated group vs. control group, at the same time point, n = 6).

Compared with saline-injected control mice, long-term administration of D-galactose and NaNO_2_ resulted in “aging” symptoms similar to clinical presentations of AD, such as sparse dry hair, flabby skin, reduced food intake, weight loss, slow response and disrupted motor activity. Reduced cognition function of senescent mice was evidenced in MWM tasks. All mice successfully learned to find the hidden platform after four days training sessions. Figure 1 shows the average escape latencies of different groups onto a hidden platform in the acquisition trials of the MWM test, with progressively shorter latency on consecutive days. Overall, there was a significant effect of day [F (3, 24) = 139.536. p = 0.000] on latency and interaction of day x group session was also observed [F(3, 24) = 2.859. p<0.05]. Compared with saline-injected control mice, remarkable memory impairment was evidenced in senescent mice on day 30, indicated by a significant prolongation of mean escape latencies (p<0.01). Learning and memory ability declined progressively and the mean escape latencies in senescent mice on day 70 were significantly prolonged compared with those of 30-day model group (p<0.05) (Table 1). There was no significant difference among pairs of latencies from any other two model groups.

**Table 1:** Comparison of the escape latencies among different groups in MWM test (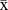
±s, n = 6).

	Mean escape latency at different time (s)			
Group	Day 1	Day 2	Day 3	Day 4
Control mice	29.42±14.16	25.35±10.87	21.32±11.28	10.73±6.41
senescent mice on day 30	77.17±22.44**	59.81±18.16**	54.91±16.13**	50.81±16.93**
senescent mice on day 40	74.12±21.27**	68.58±20.16**	63.44±18.67**	56.37±15.70**
senescent mice on day 50	87.64±15.62**	70.84±9.35**	62.39±7.28**	61.07±7.38**
senescent mice on day 60	90.5±15.04**	71.81±10.07**	63.87±8.09**	57.38±5.85**
senescent mice on day 70	97.41±10.71** #	76.61±9.19** #	75.06±7.27** #	63.55±4.17** #

**p<0.01: senescent mice versus control mice, ^#^ p<0.05: senescent mice on day 70 versus senescent mice on day 30, at the same time point.

### ^1^H MRS Analysis

**Figure 2:**
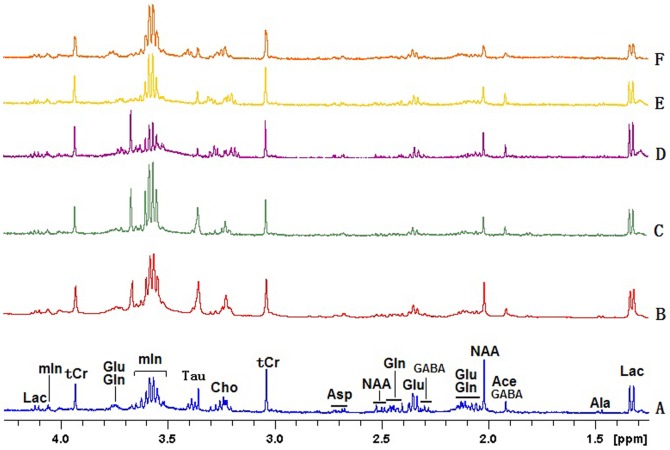
Representative *in vitro* ^1^H NMR spectra at 9.4T from control (A) and senescent mice induced with D-galactose and NaNO_2_ on day 30 (B), day 40 (C), day 50 (D), day 60 (E), and day 70 (E), respectively. Lac: lactate; Ala: alanine; Ace: acetate; GABA: g-aminobutyric acid; NAA: N-acetylaspartate; Glu: glutamate; Glu: glutamine; Asp: aspatate; tCr: total creatine (creatine and phosphocreatine); Cho:choline; mIn: myo-inositol.

**Figure 3:**
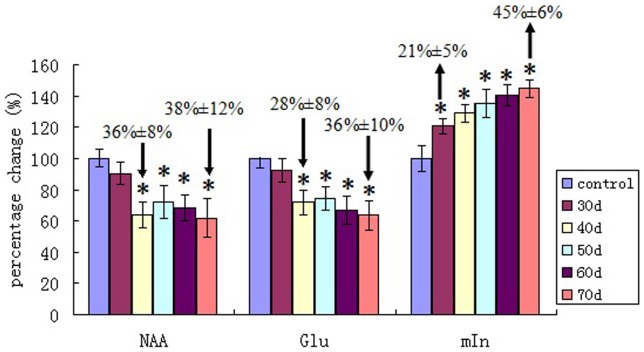
Averaged relative percentage changes of metabolites over six subjects between control mice and senescent mice (Change±s.d). *P<0.01, versus control group. NAA: N-acetylaspartate; Glu: glutamate; mIns: myo-inositol.

**Figure 4:**
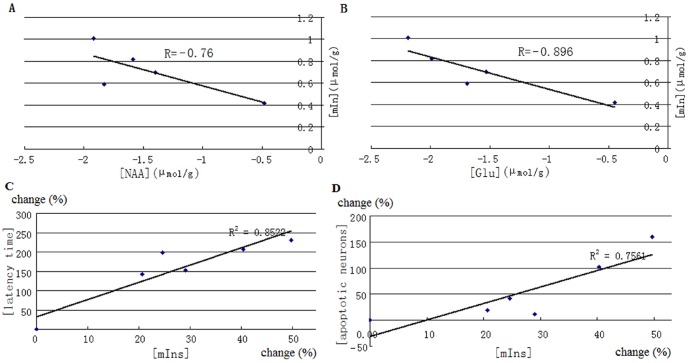
Correlation between the changes in mIns and NAA (A), mIns and Glu (B), mIns and latency time (C), mIns and apoptotic neurons (D) in senescent mice at different time point.

Figure 2 depicted a series of high quality ^1^H-NMR spectra from hippocampal extracts as evidenced by the smoothness of the baseline. The peaks at 1.33&4.12 ppm (Lactate, Lac), 1.47 ppm (Alanine, Ala), 1.90 ppm (Acetate, Ace), 2.02 ppm (N-acetylaspartate, NAA), 1.89&2.31 ppm (g-aminobutyric acid, GABA), 2.12&2.35& 3.75 ppm (Glu), 2.12&2.45&3.75 ppm (Glutamine, Gln), 2.65 ppm (Aspartate, Asp), 3.03&3.94 ppm (total creatine, tCr), 3.21 ppm (Choline, Cho), 3.42 ppm (Taurine, Tau), 3.5–3.6 &4.04 ppm (myo-inositol, mIns) were clearly visible. Concentrations of NAA, Glu, Cho, mIns, tCr and Lac were measured (Table 2). In good agreement with previous *in vivo* ^1^H MRS findings in human pre-symptomatic AD and AD transgenic mice, our *in vitro* ^1^H MRS findings at 9.4T in senescent mice revealed a significant reduction in levels of NAA and Glu, coupled with a marked increase of mIns in the hippocampus (Figure 3). Compared with the averaged concentration of NAA (5.04±0.28 µmol/g) in saline-injected control group, the mean concentration of NAA in model groups tended to be reduced on day 30, and reached significance on day 40 (reduced by 1.8±0.26 µmol/g, equivalent to 36%±8%, p<0.01). A similar change was identified in Glu, which significantly decreased by 1.7±0.35 µmol/g (equivalent to 28%±8%) on day 40, compared with that of 6.00±0.37 µmol/g in the control group. Both NAA and Glu in model mice on day 50 displayed a slight recovery but still remained significantly lower than that of the control group and continued to fall through on day 60 and day 70. The decreases in NAA and Glu were much pronounced on day 70 (−38%±12% and −36%±10% respectively, p<0.01). mIns exhibited significant elevation during early–stage of administration on day 30 (increased by 21%±5%) and increasing further up to day 70 (45%±6%). The changes of mIns appeared to be negatively correlated with the changes of NAA and Glu over time (Figures 4A, 4B), and the relative percentage changes of mIns was found to be positively correlated with the relative percentage changes of escape latencies (Figure 4C) and apoptotic neurons over time (Figure 4D). tCr, Cho and Lac in model groups showed a slight decrease along with long-term duration of administration but neither reached significance.

**Table 2:** Absolute concentrations of metabolites in the hippocampus by *in vitro* ^1^H MRS(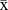
±s, μmol/g, n = 6).

		D-galactose & NaNO_2_ treated group				
Metabolite	Control	Day 30	Day 40	Day 50	Day 60	Day 70
NAA	5.04±0.28	4.56±0.32	3.21±0.26*	3.64±0.38*	3.45±0.29*	3.12±0.38*
Glu	6.00±0.37	5.55±0.42	4.31±0.35*	4.47±0.33*	4.01±0.35*	3.81±0.37*
mIns	2.03±0.17	2.45±0.12*	2.62±0.15*	2.73±0.23*	2.85±0.20*	3.04±0.17*
tCr	7.04±0.28	6.86±0.22	7.21±0.16	7.24±0.28	7.27±0.19	7.12±0.20
Lac	1.85±0.22	1.77±0.12	1.75±0.15	1.74±0.21	1.78±0.25	1.69±0.21
Cho	1.22±0.15	1.21±0.12	1.09±0.16	1.16±0.13	1.15±0.14	1.01′±0.16

NAA: N-acetylaspartate; Glu: glutamate; mIns: myo-inositol; tCr: total creatine (creatine and phosphocreatine); Lac: lactate; Cho:choline; *P<0.01, versus control mice.

### Pathomorphological Observation

**Figure 5:**
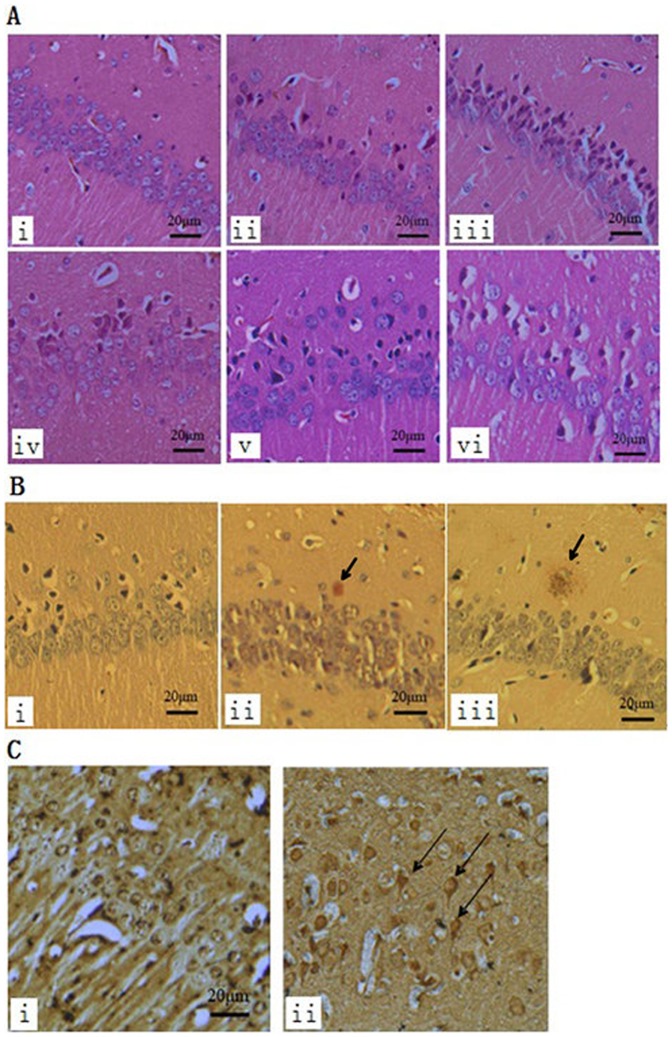
Histopathological architecture of the hippocampus. A: HE staining of the hippocampus (magnification x 400 in A–E) revealed the progression of neuronal apotosis. (i) control group: the neurons were intact and arrange tightly; (ii) model group (on day 30): some densely stained neurons were presented; (iii, iv) model group (on day 40 and day 50): long length administration of drug markedly increased the number of damaged pyramidal cells in the hippocampus; (v, vi) model group (on day 60 and day70): pyramidal neurons either presented a densely stained shrunken appearance with minimal cytoplasm or had disappeared and the cell array became sparse. B: Congo red staining of the hippocampus. (i) control group: normal cellular distribution without amyloid deposition; (ii, iii) model group (on day 60 and 70): presented a collection of orange amylaceous aggradation-amyloid deposition (arrows). C: Bielschowsky silver impregnation of the hippocampus. (i) control group: no evidence of neurofibrillary tangles; (ii) model group (on day 70): neurofibrillary tangles were presented and stained neuropil threads (arrows).

There were typical neuropathological changes in the hippocampus in senescent mice induced by D-galactose and NaNO_2_. Figure 5A showed the histopathological architecture of the hippocampus with HE staining. In the control group, pyramidal neurons were laid in three to four layers and arranged tightly and the nuclei were round, large and light stained (Figure.5A(i)). Progressive neuron loss was evidenced in senescent mice along with the prolongation of administration (Figure 5A(ii–vi)). Remarkable neuronal damage was manifested on day 60 and day 70 (Table 3) and neuronal arrays were sparse and disordered (Figure 5A (v, vi)), which were rarely observed in the age-matched control. Besides notable neuronal loss, progressive formation of amyloid accumulation (Figure 5B (iii)) and neurofibrillary tangles (Figure 5C (ii)) could also be visualized in senescent mice through Congo red staining and Bielschowsky sliver staining, respectively, and the neurons on day 70 were heavily stained (Figure 5C (ii)).

**Table 3:** Numbers of apoptotic neurons, amyloid plaques and neurofibrillary tangles in the hippocampus in different mice groups at different time point (n/mm^2^, n = 6).

Histopathological features		D-galactose & NaNO_2_ treated group				
	Control	day 30	day 40	day 50	day 60	day 70
apoptotic neurons	4.57±2.19	5.45±2.16	5.12±1.99	6.47±1.92	9.21±2.05*	11.87±1.96*
amyloid plaques	-	-	-	-	2±1.2	3±1.4
neurofibrillary tangles	-	-	-	-	-	4±1.5

Values are expressed as mean ± SD. *p<0.01: senescent mice versus control mice.

### The Relationship Between Histological Changes and Metabolite Levels

**Figure 6:**
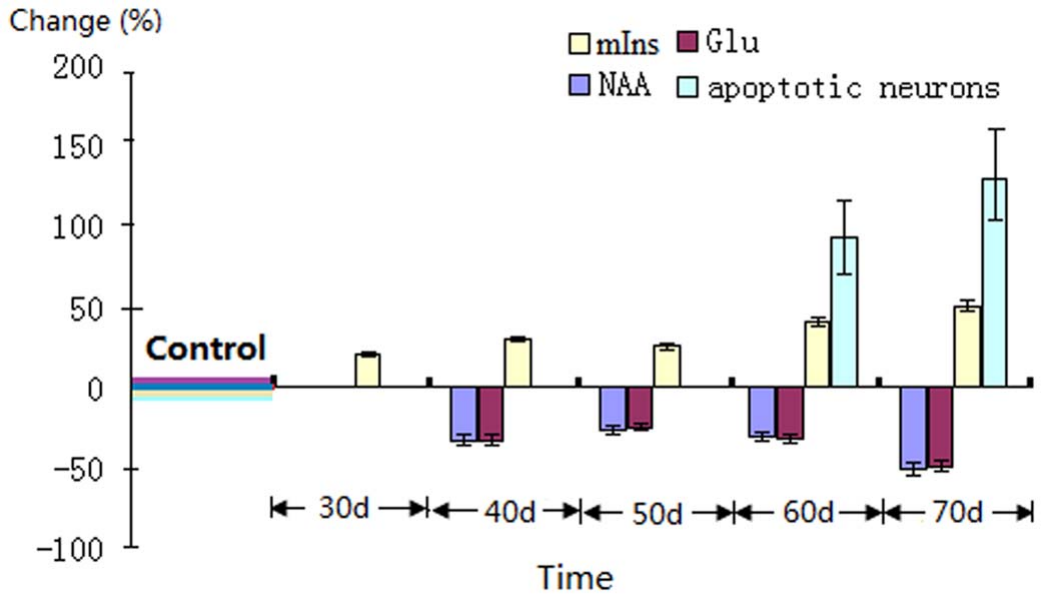
Significant changes of mIns, NAA, Glu and apoptotic neurons in senescent mice at different time point. Data (mean ± SD) are expressed as a % relative to the average control level. Changes in mIns (started on day 30), NAA and Glu (started on day 40) preceded brain neuronal degeneration (started on day 60).

Strong association was observed between the *in vitro* ^1^H MRS findings and histopathological observations. Changes in mIns (started on day 30), NAA and Glu (started on day 40) occurred earlier than neuronal degeneration (started on day 60), and elevated mIns occurred prior to the decreased NAA and Glu during aging progression (Figure 6). Decreasing NAA and Glu were accompanied by morphological abnormalities including neuronal loss, amyloid plaques and neurofibrillary tangles as evidenced in hippocampal subdivisions in senescent mice.

## Discussion

Knowledge on the underlying pathology behind AD has been gained greatly in recent years, thanks to the studies of animal models. There have been various animal models of human AD, but they fail to represent AD comprehensively and accurately. Transgenic mouse models mimicking Alzheimer's disease are useful for studying disease mechanisms [32], most of which were based on the overexpression of mutated forms of human amyloid-β protein precursor (AβPP) or in combination with mutated human presenilin 1 (PS1) or 2 (PS2) genes [28], presenting typical histopathological features of AD. However, most of them did not show the complete phenotype of human AD including neurofibrillary tangles and massive neuronal loss [32]. Long-term administration of D-galactose was reported to induce senescent syndromes in animals similar to those seen in aging humans [40], [41], [42], [43]. Injection of NaNO_2_ could result in memory-consolidating disability, probably because the nitric salt changes normal haemoglobin into methaemoglobin, which reduces the oxygen-carrying capacity of the blood and causes hypoxia and impaired consciousness in mice [40]. Experimentally-induced senescent mice used in this study was characterized by aging symptoms, an early-onset memory dysfunction (Figure 1, Table 1) and pathological abnormalities including neuronal loss, amyloid plaques and neurofibrillary tangles (Figure 5, Table 3). This animal model has facilitated the evaluation of early biomarkers for AD, allowing researchers to perform longitudinal studies starting before the onset of the pathological lesions.

There is a considerable interest in identifying early neurochemical biomarkers for prodromal AD using ^1^H-MRS. Numerous in vivo ^1^H MRS studies in human pre-symptomatic AD [10], [11], [12], [13], [19], [20], [21], [22], [23], [24] and AD transgenic mice [29], [30], [31] have reported decreased brain Glu and NAA levels and elevated mIns, and we too observe similar changes of hippocampal neurochemical profile in the senescent mice, based on *in vitro* ^1^H MRS on the perchloric acid extractions of the hippocampus. As expected, changes in mIns (started on day 30), NAA and Glu (started on day 40) occurred earlier than neuronal degeneration (started on day 60). In addition, Elevated mIns occurred prior to the decreased NAA and Glu during the progression of aging (Figure 6).

NAA at 2.02 ppm is an amino acid derivative synthesized in the mitochondria in neural cells, and also involved in the synthesis of myelin. It has been regarded as a very specific marker for viable neurons, axons and dendrites. Decreased brain NAA concentration may reflect a combination of neuronal atrophy, axonal loss, decreased neural metabolism, reduced myelination and loss of dendritic structures [44]. Several *in vivo* ^1^H MRS studies have suggested that NAA could be used to discriminate normal aging and pathologic dementia effectively [45], [46], [47], [48]. A consistent NAA decline in subjects with AD and mild cognitive impairment has been reported [11], [30], [31], [46], [48], [49], [50]. In good agreement with previous *in vivo* ^1^H MRS findings, our *in vitro* ^1^H MRS study of senescent mice also observed decreased hippocampal NAA, which reduced significantly from a lower starting point in the early stages of AD-like lesion induction on day 40 and decreased further up to day 70, accompanied by progressive morphological abnormalities including neuronal loss, amyloid plaques and neurofibrillary tangles. These findings validated the concept that NAA depletion could reflect the pathologic progression of neuronal degeneration in AD.

Glu is the most abundant excitatory neurotransmitter in the central nervous system (CNS), involving in cognitive and emotional activities. Previous *in vivo* ^1^H MRS studies in human brain have depicted a progressive decrease in hippocampal Glu from cognitive control to mild cognitive impairment (MCI) to full-blown AD [13], [24], [29], [50]. However, only relative decreases in the sum of Glu and glutamine (Gln) over tCr were obtained, due to the reason that the metabolite resonances of Glu and Gln closely overlap in the spectral domain at lower field strengths. The high magnetic field at 9.4T used in current study, provides increased signal-to-noise ratio and spectral resolution, and allows reliable absolute quantification of Glu. Consistent with previous *in vivo* ^1^H MRS studies, our *in vitro* ^1^H MRS findings in senescent mice revealed a progressive Glu reduction in response to prolonged administration of D-galactose and NaNO_2_. Decreased Glu may reflect a loss of glutamatergic neurons or decreased glutamatergic synthesis [50], which contributes to the memory dysfunction in AD. A histological study has found an 80% decrease in Glu in the hippocampal formation of subjects with AD, compared to controls [51].

Brain mIns exists in the astrocytes and has been proposed to be a marker of gliosis or microglial activation [52], based on observations on cultured glial tumor cells [53]. Many *in vivo* ^1^H MRS studies have found increased mIns levels in the temporal, parietal and occipital lobes in AD patients [20], [31], [45], [54]. In this study, mIns exhibited significant elevation during early–stage of administration on day 30 and remained elevated during prolonged chemical induction. Elevated mIns levels might be related to the activation and reactive hyperplasia of astrocytes [31], [55]. It could be also hypothesized that there is a strong glial activation due to increased plaque deposition in AD [56]. However, Dedeoglu et al [57] did not find any significant difference in mIns levels between 10 and 12-month-old APP mice and wild type mice. These varied mIns results may be influenced by variations of different mouse AD models.

The exact mechanism of mIns abnormality prior to NAA and Glu remains unclear, but evidence suggested that astrocytic response (or glial proliferation) may have been enhanced, preceding significant neuronal loss or mitochondrial dysfunction, to prevent the formation of senile plaques and to maintain the microenvironment of CNS cells in the early phase of AD [31], [55]. Chen et al performed GFAP staining and found that there were activated and hyperplastic astrocytes in the frontal cortex and hippocampus in 3-month-old AD mice, while no Aβplaques were detected and neurons were also not damaged [31]. Quantification of glial activation and the exploration of the potential value of mIns for early diagnosis of AD would be the subject of further studies in our laboratory.

## Conclusions

In good agreement with previous *in vivo* ^1^H MRS findings, our *in vitro* ^1^H MRS at 9.4T observed decreased NAA and Glu and increased mIns in the hippocampus of senescent mice (prodromal stage AD) induced by D-galactose and NaNO_2_. Changes in mIns, NAA and Glu preceded neuronal degeneration. Elevated mIns occured prior to the decreased NAA and Glu during the aging process. Decreased NAA and Glu can be interpreted in terms of neuronal degeneration and glutamatergic activity dysfunction that may contribute to the pathophysiological mechanisms underlying AD. Further experiments using different AD animal models need to be performed to explore the potential value of mIns in the early diagnosis of AD, to verify whether glial cell proliferation occurs earlier than the neuronal changes.

## References

[1] Ferri CP, Prince M, Brayne C, Brodaty H, Fratiglioni L, et al (2005) Global prevalence of dementia: a Delphi consensus study. Lancet 366: 2112–2117. 16360788 10.1016/S0140-6736(05)67889-0 PMC2850264

[2] Villemagne VL, Rowe CC, Macfarlane S, Novakovic KE, Masters CL (2005) Imaginem oblivionis: the prospects of neuroimaging for early detection of Alzheimer's disease. J Clin Neurosci 12: 221–230. 15851069 10.1016/j.jocn.2004.03.011

[3] Blennow K, de Leon MJ, Zetterberg H (2006) Alzheimer's disease. Lancet 368: 387–403. 16876668 10.1016/S0140-6736(06)69113-7

[4] Terry AV, Callahan PM, Hall B, Webster SJ (2011) Alzheimer's disease and age-related memory decline (preclinical). Pharmacol Biochem Behav 99: 190–210. 21315756 10.1016/j.pbb.2011.02.002 PMC3113643

[5] Jack CR Jr, Petersen RC, Xu YC, O'Brien PC, Smith GE, et al (1999) Prediction of AD with MRI-based hippocampal volume in mild cognitive impairment. Neurology 52: 1397–1403. 10227624 10.1212/wnl.52.7.1397 PMC2730146

[6] Rabinovici GD, Seeley WW, Kim EJ, Gorno-Tempini ML, Rascovsky K, et al (2007) Distinct MRI atrophy patterns in autopsy-proven Alzheimer's disease and frontotemporal lobar degeneration. Am J Alzheimers Dis Other Demen 22: 474–488. 18166607 10.1177/1533317507308779 PMC2443731

[7] Klunk WE, Engler H, Nordberg A, Wang Y, Blomqvist G, et al (2004) Imaging brain amyloid in Alzheimer's disease with Pittsburgh Compound-B. Ann Neurol 55: 306–319. 14991808 10.1002/ana.20009

[8] Rowe CC, Ng S, Ackermann U, Gong SJ, Pike K, et al (2007) Imaging beta-amyloid burden in aging and dementia. Neurology 68: 1718–1725. 17502554 10.1212/01.wnl.0000261919.22630.ea

[9] Newberg AB, Wintering NA, Plossl K, Hochold J, Stabin MG, et al (2006) Safety, biodistribution, and dosimetry of 123I-IMPY: a novel amyloid plaque-imaging agent for the diagnosis of Alzheimer's disease. J Nucl Med 47: 748–754. 16644743

[10] Kantarci K, Weigand SD, Petersen RC, Boeve BF, Knopman DS, et al (2007) Longitudinal 1H MRS changes in mild cognitive impairment and Alzheimer's disease. Neurobiol Aging 28: 1330–1339. 16860440 10.1016/j.neurobiolaging.2006.06.018 PMC2766807

[11] Dixon RM, Bradley KM, Budge MM, Styles P, Smith AD (2002) Longitudinal quantitative proton magnetic resonance spectroscopy of the hippocampus in Alzheimer's disease. Brain 125: 2332–2341. 12244089 10.1093/brain/awf226

[12] Adalsteinsson E, Sullivan EV, Kleinhans N, Spielman DM, Pfefferbaum A (2000) Longitudinal decline of the neuronal marker N-acetyl aspartate in Alzheimer's disease. Lancet 355: 1696–1697. 10905250 10.1016/s0140-6736(00)02246-7

[13] Rupsingh R, Borrie M, Smith M, Wells JL, Bartha R (2011) Reduced hippocampal glutamate in Alzheimer disease. Neurobiol Aging 32: 802–810. 19501936 10.1016/j.neurobiolaging.2009.05.002

[14] Kantarci K, Petersen RC, Boeve BF, Knopman DS, Tang-Wai DF, et al (2004) 1H MR spectroscopy in common dementias. Neurology 63: 1393–1398. 15505154 10.1212/01.wnl.0000141849.21256.ac PMC2766798

[15] Metastasio A, Rinaldi P, Tarducci R, Mariani E, Feliziani FT, et al (2006) Conversion of MCI to dementia: Role of proton magnetic resonance spectroscopy. Neurobiol Aging 27: 926–932. 15936850 10.1016/j.neurobiolaging.2005.05.002

[16] Tkac I, Gruetter R (2005) Methodology of H NMR Spectroscopy of the Human Brain at Very High Magnetic Fields. Appl Magn Reson 29: 139–157. 20179773 10.1007/BF03166960 PMC2825674

[17] Lin Y, Stephenson MC, Xin L, Napolitano A, Morris PG (2012) Investigating the metabolic changes due to visual stimulation using functional proton magnetic resonance spectroscopy at 7 T. J Cereb Blood Flow Metab. 32: 1484–1495. 10.1038/jcbfm.2012.33 PMC3421086 22434070

[18] Stephenson MC, Gunner F, Napolitano A, Greenhaff PL, Macdonald IA, et al (2011) Applications of multi-nuclear magnetic resonance spectroscopy at 7T. World J Radiol 3: 105–113. 21532871 10.4329/wjr.v3.i4.105 PMC3084434

[19] Huang W, Alexander GE, Chang L, Shetty HU, Krasuski JS, et al (2001) Brain metabolite concentration and dementia severity in Alzheimer's disease: a (1)H MRS study. Neurology 57: 626–632. 11524470 10.1212/wnl.57.4.626

[20] Herminghaus S, Frolich L, Gorriz C, Pilatus U, Dierks T, et al (2003) Brain metabolism in Alzheimer disease and vascular dementia assessed by in vivo proton magnetic resonance spectroscopy. Psychiatry Res 123: 183–190. 12928106 10.1016/s0925-4927(03)00071-4

[21] Valenzuela MJ, Sachdev P (2001) Magnetic resonance spectroscopy in AD. Neurology 56: 592–598. 11261442 10.1212/wnl.56.5.592

[22] Kantarci K (2007) 1H magnetic resonance spectroscopy in dementia. Br J Radiol 80 Spec No 2: S146–152. 10.1259/bjr/60346217 18445744

[23] Schuff N, Capizzano AA, Du AT, Amend DL, O'Neill J, et al (2002) Selective reduction of N-acetylaspartate in medial temporal and parietal lobes in AD. Neurology 58: 928–935. 11914410 10.1212/wnl.58.6.928 PMC1851674

[24] Fayed N, Modrego PJ, Rojas-Salinas G, Aguilar K (2013) Brain glutamate levels are decreased in Alzheimer's disease: a magnetic resonance spectroscopy study. Am J Alzheimers Dis Other Demen 26: 450–456. 10.1177/1533317511421780 PMC10845671 21921084

[25] Pfefferbaum A, Adalsteinsson E, Spielman D, Sullivan EV, Lim KO (1999) In vivo spectroscopic quantification of the N-acetyl moiety, creatine, and choline from large volumes of brain gray and white matter: effects of normal aging. Magn Reson Med 41: 276–284. 10080274 10.1002/(sici)1522-2594(199902)41:2<276::aid-mrm10>3.0.co;2-8

[26] Krishnan KR, Charles HC, Doraiswamy PM, Mintzer J, Weisler R, et al (2003) Randomized, placebo-controlled trial of the effects of donepezil on neuronal markers and hippocampal volumes in Alzheimer's disease. Am J Psychiatry 160: 2003–2011. 14594748 10.1176/appi.ajp.160.11.2003

[27] Parnetti L, Tarducci R, Presciutti O, Lowenthal DT, Pippi M, et al (1997) Proton magnetic resonance spectroscopy can differentiate Alzheimer's disease from normal aging. Mech Ageing Dev 97: 9–14. 9223122 10.1016/s0047-6374(97)01877-0

[28] Hall AM, Roberson ED (2011) Mouse models of Alzheimer's disease. Brain Res Bull 88: 3–12. 22142973 10.1016/j.brainresbull.2011.11.017 PMC3546481

[29] Marjanska M, Curran GL, Wengenack TM, Henry PG, Bliss RL, et al (2005) Monitoring disease progression in transgenic mouse models of Alzheimer's disease with proton magnetic resonance spectroscopy. Proc Natl Acad Sci U S A 102: 11906–11910. 16091461 10.1073/pnas.0505513102 PMC1188012

[30] Chen SQ, Cai Q, Shen YY, Wang PJ, Teng GJ, et al (2012) Age-related changes in brain metabolites and cognitive function in APP/PS1 transgenic mice. Behav Brain Res 235: 1–6. 22828014 10.1016/j.bbr.2012.07.016

[31] Chen SQ, Wang PJ, Ten GJ, Zhan W, Li MH, et al (2009) Role of myo-inositol by magnetic resonance spectroscopy in early diagnosis of Alzheimer's disease in APP/PS1 transgenic mice. Dement Geriatr Cogn Disord 28: 558–566. 20093832 10.1159/000261646 PMC2837893

[32] Mlynarik V, Cacquevel M, Sun-Reimer L, Janssens S, Cudalbu C, et al (2012) Proton and phosphorus magnetic resonance spectroscopy of a mouse model of Alzheimer's disease. J Alzheimers Dis 31 Suppl 3S87–99. 22451319 10.3233/JAD-2012-112072

[33] Bayer TA, Breyhan H, Duan K, Rettig J, Wirths O (2008) Intraneuronal beta-amyloid is a major risk factor–novel evidence from the APP/PS1KI mouse model. Neurodegener Dis 5: 140–142. 18322372 10.1159/000113684

[34] Howlett DR, Richardson JC, Austin A, Parsons AA, Bate ST, et al (2004) Cognitive correlates of Abeta deposition in male and female mice bearing amyloid precursor protein and presenilin-1 mutant transgenes. Brain Res 1017: 130–136. 15261108 10.1016/j.brainres.2004.05.029

[35] Forster DM, James MF, Williams SR (2012) Effects of Alzheimer's disease transgenes on neurochemical expression in the mouse brain determined by (1)H MRS in vitro. NMR Biomed 25: 52–58. 22241671 10.1002/nbm.1712

[36] Barton SJ, Howe FA, Tomlins AM, Cudlip SA, Nicholson JK, et al (1999) Comparison of in vivo1H MRS of human brain tumours with1H HR-MAS spectroscopy of intact biopsy samples in vitro. Magnetic Resonance Materials in Physics, Biology and Medicine 8: 121–128. 10.1007/BF02590529 10456375

[37] Fischer AH, Jacobson KA, Rose J, Zeller R (2008) Hematoxylin and eosin staining of tissue and cell sections. CSH Protoc 2008: pdb prot4986. 10.1101/pdb.prot4986 21356829

[38] Carson FL, Kingsley WB (1980) Nonamyloid green birefringence following Congo red staining. Arch Pathol Lab Med 104: 333–335. 6990892

[39] Litchfield S, Nagy Z (2001) New temperature modification makes the Bielschowsky silver stain reproducible. Acta Neuropathol 101: 17–21. 11194935 10.1007/s004010000248

[40] Fang F, Liu G (2007) A novel cyclic squamosamide analogue compound FLZ improves memory impairment in artificial senescence mice induced by chronic injection of D-galactose and NaNO2. Basic Clin Pharmacol Toxicol 101: 447–454. 17971066 10.1111/j.1742-7843.2007.00138.x

[41] Chu J LL (2003) D-galactose induced brain senescence model and its mechanism. Chin J Rehabil Theory Practice 9: 521–522.

[42] Ho SC, Liu JH, Wu RY (2003) Establishment of the mimetic aging effect in mice caused by D-galactose. Biogerontology 4: 15–18. 12652185 10.1023/a:1022417102206

[43] Wei H, Li L, Song Q, Ai H, Chu J, et al (2005) Behavioural study of the D-galactose induced aging model in C57BL/6J mice. Behav Brain Res 157: 245–251. 15639175 10.1016/j.bbr.2004.07.003

[44] Birken DL, Oldendorf WH (1989) N-acetyl-L-aspartic acid: a literature review of a compound prominent in 1H-NMR spectroscopic studies of brain. Neurosci Biobehav Rev 13: 23–31. 2671831 10.1016/s0149-7634(89)80048-x

[45] Jones RS, Waldman AD (2004) 1H-MRS evaluation of metabolism in Alzheimer's disease and vascular dementia. Neurol Res 26: 488–495. 15265265 10.1179/016164104225017640

[46] Jessen F, Traeber F, Freymann N, Maier W, Schild HH, et al (2005) A comparative study of the different N-acetylaspartate measures of the medial temporal lobe in Alzheimer's disease. Dement Geriatr Cogn Disord 20: 178–183. 16024934 10.1159/000087095

[47] Ackl N, Ising M, Schreiber YA, Atiya M, Sonntag A, et al (2005) Hippocampal metabolic abnormalities in mild cognitive impairment and Alzheimer's disease. Neurosci Lett 384: 23–28. 15905028 10.1016/j.neulet.2005.04.035

[48] den Heijer T, Sijens PE, Prins ND, Hofman A, Koudstaal PJ, et al (2006) MR spectroscopy of brain white matter in the prediction of dementia. Neurology 66: 540–544. 16505309 10.1212/01.wnl.0000198256.54809.0e

[49] Oberg J, Spenger C, Wang FH, Andersson A, Westman E, et al (2008) Age related changes in brain metabolites observed by 1H MRS in APP/PS1 mice. Neurobiol Aging 29: 1423–1433. 17434239 10.1016/j.neurobiolaging.2007.03.002

[50] Haris M, Nath K, Cai K, Singh A, Crescenzi R, et al (2013) Imaging of glutamate neurotransmitter alterations in Alzheimer's disease. NMR Biomed 26: 386–391. 23045158 10.1002/nbm.2875 PMC3556355

[51] Hyman BT, Van Hoesen GW, Damasio AR (1987) Alzheimer's disease: glutamate depletion in the hippocampal perforant pathway zone. Ann Neurol 22: 37–40. 2443073 10.1002/ana.410220110

[52] Lazeyras F, Charles HC, Tupler LA, Erickson R, Boyko OB, et al (1998) Metabolic brain mapping in Alzheimer's disease using proton magnetic resonance spectroscopy. Psychiatry Res 82: 95–106. 9754452 10.1016/s0925-4927(98)00010-9

[53] Brand A, Richter-Landsberg C, Leibfritz D (1993) Multinuclear NMR studies on the energy metabolism of glial and neuronal cells. Dev Neurosci 15: 289–298. 7805581 10.1159/000111347

[54] Moats RA, Ernst T, Shonk TK, Ross BD (1994) Abnormal cerebral metabolite concentrations in patients with probable Alzheimer disease. Magn Reson Med 32: 110–115. 8084225 10.1002/mrm.1910320115

[55] Kimura N, Takahashi M, Tashiro T, Terao K (2006) Amyloid beta up-regulates brain-derived neurotrophic factor production from astrocytes: rescue from amyloid beta-related neuritic degeneration. J Neurosci Res 84: 782–789. 16862545 10.1002/jnr.20984

[56] Coimbra A, Williams DS, Hostetler ED (2006) The role of MRI and PET/SPECT in Alzheimer's disease. Curr Top Med Chem 6: 629–647. 16712496 10.2174/156802606776743075

[57] Dedeoglu A, Choi JK, Cormier K, Kowall NW, Jenkins BG (2004) Magnetic resonance spectroscopic analysis of Alzheimer's disease mouse brain that express mutant human APP shows altered neurochemical profile. Brain Res 1012: 60–65. 15158161 10.1016/j.brainres.2004.02.079

